# Effects of macroporous monofilament mesh on infection in a contaminated field

**DOI:** 10.1007/s00423-014-1225-3

**Published:** 2014-08-29

**Authors:** Kamil Bury, Maciej Śmietański, Bigda Justyna, Piotr Gumiela, Anna Irmina Śmietańska, Radosław Owczuk, Łukasz Naumiuk, Alfred Samet, J. Paradziej-Łukowicz

**Affiliations:** 1 Department of Cardiac and Vascular Surgery, Medical University of Gdansk, Gdansk, Poland; 2 Department of General and Vascular Surgery, Ceynowa Hospital in Wejherowo, Wejherowo, Poland; 3 Department of General, Endocrine and Transplant Surgery, Medical University of Gdansk, Gdansk, Poland; 4 Tri-City Central Animal Laboratory – Research and Service Centre, Medical University of Gdansk, Gdansk, Poland; 5 Department of Medical Microbiology, Medical University of Gdansk, Gdansk, Poland; 6 Department of Anesthesiology and Intensive Care, Medical University of Gdansk, Gdansk, Poland

**Keywords:** Mesh, Hernia, Infection, Experimental, Rat model

## Abstract

**Background:**

The aim of this study was to evaluate whether the type of the mesh and proper surgical technique can influence the outcome of a tension-free hernia repair in a contaminated filed.

**Materials and methods:**

This study was based on the model of bacterial peritonitis in rats induced with a mixture composed of *Escherichia coli* and *Bacteroides fragilis*. Two animals were used as a control group without induced peritonitis and 10 animals with mesh implanted inside of the peritoneal cavity. For the 20 animals in the studied group, bacterial fluid was applied into the abdominal cavity together with the mesh implantation. In 10 cases, the mesh was fixed flatly upon the surface of the peritoneum; in the other 10, the mesh was rolled and then fixed within the peritoneal cavity. After 5 weeks, the animals were operated on again, and the meshes, the peritoneal fluid and, if present, any granulomas were taken for bacterial cultivation.

**Results:**

The results of the bacterial cultivation of the material from the control group (without mesh) and from the rats with flatly fixed mesh were almost completely negative (0/10 and 1/10, respectively). In 9 out of 10 rats that were exposed to the rolled mesh for 5 weeks, the colonisation of meshes with both *B. fragilis* and *E. coli* was found (*p* < 0.0198).

**Conclusions:**

When properly fixed, flat mesh, even in a contaminated field, may allow for a proper mesh healing and does not influence the ability to cure bacterial peritonitis in an animal model. A bad surgical technique, such as inadequately positioned or rolled mesh, may cause persistent peritoneal bacteraemia.

## Introduction

Hernia repair operations are among the most frequently performed procedures in surgical departments. Abdominal hernias, both primary and incisional, continue to represent a serious clinical problem. The risk of a incisional hernia is estimated to be approximately 11 % [1] for treatments involving a median incision. The data obtained from the literature show that the use of a synthetic implant significantly reduces the hernia recurrence rate. However, mesh-related complications may still occur in 10–15 % of the patients in cases where bacteria are present in the operative field. These complications can include surgical site infection (SSI), a fistula between the mesh and the intestine or a significant loss of skin, which often forces the surgeon to remove the implanted mesh [2, 3]. In the case of mesh implantation in an infected surgical field, the percentage of mesh removal can be as high as 90 % [4]. In the case of mesh explantation, the authors do not discuss the method of its fixation [5–7]. Hence, recent research recommends to stop using synthetic mesh in the case of any operative field contamination [8]. Recent studies associated with infected mesh do not have information regarding the type of implants, which implants should be avoided or descriptions of the surgical techniques used. The currently used implants, which have macropore monofilament characteristics and are made from a material characterised by a significantly lower susceptibility to colonisation and infection, should in theory not contribute to the persistence of chronic infection in the operated area [9–11].

The aim of this study was to evaluate whether the use of a macroporous mesh implant in an infected area will sustain the infection. Furthermore, whether an incorrect technique of mesh implantation (i.e., the formation of dead space by bending and folding the material) affects the maintenance of the infection was also examined because an infection could result in the need to remove the synthetic material.

## Materials and methods

### The study protocol

The study protocol was approved by the Bioethics Committee of the Medical University of Gdansk (proof number 23/2007).

The study used male Wistar rats weighing 280 to 340 g. The rats were housed under consistent living conditions (day/night cycle of 12 h at 25 °C) at the Tri-City Central Animal Laboratory – Research and Service Centre MUG during the entire study. They were under the care of a veterinarian and had constant access to water and food. Throughout the study, no antibiotics were applied. According to the study protocol, the animals were randomly divided into four groups: A—the control group, with the same mixture of bacteria intraperitoneally; B—with flat mesh fixed intraperitoneally without a mixture of bacteria; C—with flat mesh fixed intraperitoneally with a mixture of bacteria; and D—with rolled mesh fixed intraperitoneally with a mixture of bacteria. The number in each of the groups, to obtain statistical significance, was set at 10 individuals in groups B, C and D. Number of rats in group A was two, according to the requirements of the bioethics committee. (According to Polish Law, the verification of a previously proven protocol, as in the study proposed by Bosscha [9], cannot be performed on more than two individuals).

The protocol was based on a model for the self-healing of bacterial peritonitis in rats described by Bosscha [9]. In our study, the mixture of bacterial strains were *Escherichia* coli (ATCC 25922, Microbiologics, Inc. North St. Cloud, Minnesota USA) and *Bacteroides fragilis* (ATCC 25285, Microbiologics, Inc. North St. Cloud, MN, USA). The mesh used in the study was composite polypropylene-poliglecaprone mesh (Ultra-Pro, Ethicon Inc., Hamburg, Germany). This implant has a macroporous structure (the pores have a diameter of 3 mm) and is manufactured from a monofilament material. For fixing the implant, a monofilament polypropylene suture with a thickness of 5-0 was used.

The experiment was conducted under sterile conditions. The rats were anaesthetised with isoflurane and bupivacaine (0.05 mg/kg administered subcutaneously). The abdomen was then shaved and washed with Octenisept. A 4-cm long incision in the midline gave good access to the peritoneal cavity. The sterile mesh was cut into equal 2 cm × 4 cm pieces, which were placed intraperitoneally on the side of the abdomen according to the study protocol and the result of the randomisation (groups B and C received flat mesh and group D received rolled mesh). Then, using a sterile syringe, a mixture of bacteria (groups A, C and D) were administered directly into the mesh. In group B, the same amount of sterile saline was administered. The laparotomy was closed with a continuous single layer coating with Prolen 5-0 suture, and the skin was closed with a single 4-0 suture. The rats were then held in the cages for 5 weeks.

After a period of 5 weeks, the rats were killed with an injection of Morbital. Upon death, the laparotomy was performed, and the mesh was evaluated macroscopically. Fluid was collected from the peritoneum and a scrap of the peritoneal mesh was taken for microbial research. The study was carried out in the Department of Medical Microbiology, Medical University of Gdansk.

### Statistical analysis

The sample size was calculated to detect a difference between number of infections in particular groups at a minimal level of 20 %. Statistical analyses were conducted using Fisher’s exact test with Freeman-Halton modification with statistical software Statistica 8.0 PL (Polish version) (StatSoft, Tulsa, OK, USA).

## Results

All the animals with induced peritonitis (groups A, C and D) presented clinical signs of peritonitis and bacterial infection. They presented a loss of appetite, weakness, accelerated respiration and an accelerated heart rate. All animals, regardless of the severity of the symptoms, survived the entire 5-week period of observation.

The results of both the aerobic and anaerobic cultures of the peritoneal fluid from the rats in the control group (A) were negative.

**Fig. 1:**
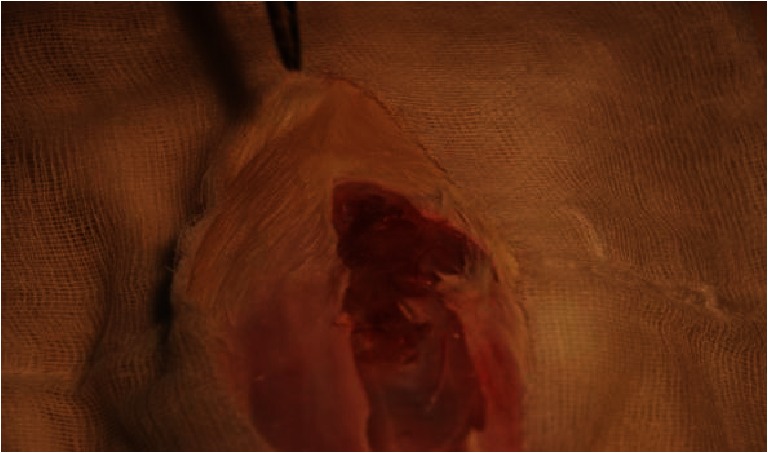
Granuloma formation on the surface of peritoneum

In group B, no cultured bacteria was detected on the mesh or in the peritoneal cavity and no granulomas were found in any of the rats. The presence of stiff adhesions in the peritoneum was noticed between the bowels and the mesh. Surgical site infections involving the skin were present in two cases; the aerobic cultures revealed the presence of *E. coli* bacteria (up to ++). In the same two individuals, granuloma formation was observed on the surface of the peritoneum in line of surgical incision (Fig. 1). The infected granulomas were cultured anaerobically to reveal the presence of *E. coli* (up to ++) and *B. fragilis* (single colonies) in the two cases.

**Fig. 2:**
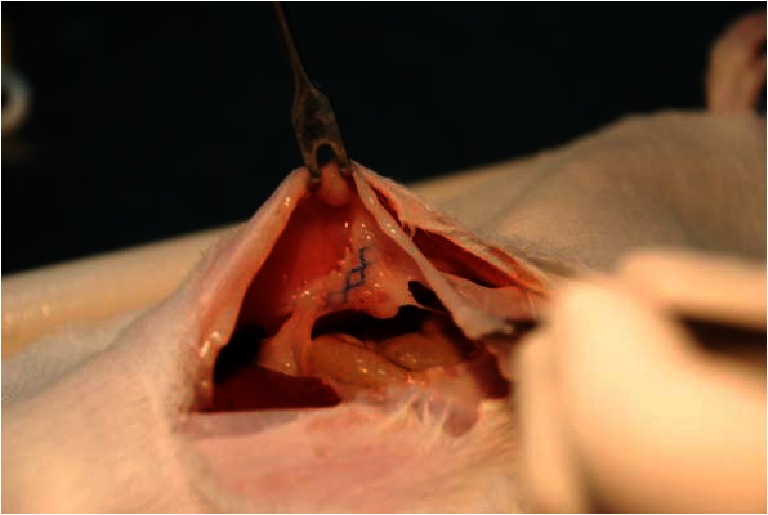
Mesh adhesions to the bowel loops or to the omentum

Among the subjects in the study group C, bacterial colonisation of the mesh was found in one individual case, which had both aerobic and anaerobic *E. coli* cultures (up to +). A macroscopic evaluation of the mesh revealed a single small abscess at the suture site with the absence of any signs of infection in the flat area of the mesh. In this same individual case, the presence of two granulomas was revealed. Aerobic cultures revealed the presence of *E. coli* bacteria (up to +); the anaerobic cultures were negative. Another individual, number 7 in group C, revealed the presence of granulomas; the aerobic and anaerobic cultures demonstrated the presence of *E. coli* (up to +). No colonisation of the mesh was noticed. A few mesh adhesions to the bowel loops or to the omentum were present (Fig. 2).

**Fig. 3:**
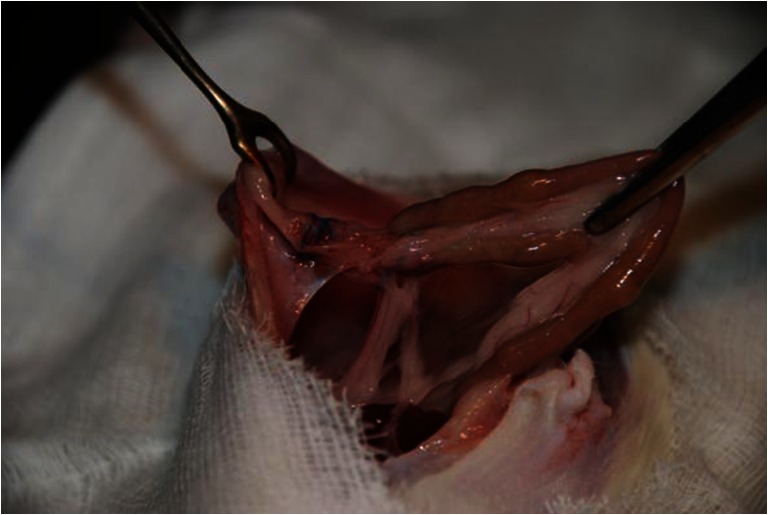
Formation of the organised abscesses

In 7 out of 10 rats in group D, bacteria that colonise the surface of a synthetic material were noted in the cultures. In this group, *E. coli* strains were grown (up to +++) using material from five individuals, and *B. fragilis* (+++) was grown from one individual. Two resulted in no growth of either *E. coli* or *B. fragilis*. Other strains were also detected: *Proteus mirabilis* (single colonies), *Staphylococcus aureus* (++), *Morganella morganii* (+) and *Streptococcus alfa* (+) haemolytic, both in aerobic and anaerobic cultures. These individuals also presented organised abscesses (Fig. 3). Table 1 summarises the aggregate results.

**Table 1:** Summarises the aggregate results

Group	Mesh cultivation	Infection in other bacterial cultures
Control group—flat mesh + saline (group B)	0/10	2/10
Flat mesh + bacteria (group C)	1/10**	2/10
Rolled mesh + bacteria (group D)	7/10**	4/10

**p = 0.0198

## Discussion

Complications may occur during repair operations that use mesh, and in the world literature, the number of infections was found to be 5–30 % for laparoscopic surgeries and up to 34 % for surgeries using a classical access method [12]. The frequency of infection has been reported to be at different levels in the available literature. Yerdel and colleagues reported four infectious complications among 280 patients undergoing inguinal hernia repair (1.4 %); in three patients, it was necessary to remove the infected mesh [13]. Other authors have reported results of up to 12.6 % for polypropylene mesh and Mersilene among a group of 63 patients undergoing surgery for a incisional hernia [14].

Because of the risk of an infection that results in the need for implant removal, some authors are proposing to use the protocols used in major orthopaedic surgery to prevent the infection of the synthetic material [15]. These activities include the proper cleaning of the operating field, frequent changes of gloves and no double-patient occupancy. In addition, many studies recommend the use of prophylactic antibiotics in each hernia surgery using a mesh implant [16]. However, this point of view has not been confirmed in meta-analysis [17].

The solution to the problem of persistent infection in synthetic materials in recent decades was to introduce biological meshes. The material consists of a collagen matrix that is inert and, while the material is undergoing remodelling, is resistant to infection. Clinical experimentation has demonstrated the possibility of using these implants in a contaminated operating field [18, 19]. However, the assumption that the matrix is replaced by the tissue of a patient raises a fundamental question. It has been proven that the hernia is a symptomatic disease of the connective tissue based on genetic mutations [20]. Replacement of the collagen implant by the defective collagen matrix of the patient may lead to a recurrence of the hernia. A recently conducted, multicentre trial, LAPSIS [21], confirms these suppositions, as do the studies published by Blatnik [22] and Gupte [23]. Therefore, it may be that biological implants grafted to the infected or contaminated surgical site are only an interim solution to the problem [24]. Furthermore, many authors have noted that there is little evidence supporting the safety of biologic grafts in a contaminated or infected field [24].

A persistent inflammatory reaction to a synthetic implant is related to the formation of a bacterial biofilm. This was initially described by Darouiche [25]. In the study, the authors noted that insufficient healing of the mesh to the host tissues is caused by a biofilm [26]. Mesh implantation, which should be treated as the introduction of a foreign body, initiates a sequence of inflammation events that begins with an acute inflammatory reaction and ends with the process of chronic adhesion [27, 28]. This process can last many years and takes the form of a subclinical process manifesting as a minor ailments of pain [27, 29]. It has also been shown that the polyfilament implants cause a much greater immune response than the monofilament mesh and, in accordance with the exposed surface of the material, favour the formation of a bacterial biofilm [27, 30, 31].

Another important factor is the pore size of the mesh. The importance of pore size is based on the assumption that bacteria penetrate freely through the microporous mesh (pores less than 10 μm). For macrophages, the micropores act as a filter that prevents effective phagocytosis [32]. The conclusion of Falgas’ study led to the introduction of a monofilament lightweight mesh containing less polypropylene into broad surgical practice [33]. Recently published papers by Sanders [34] and Deerenberg [35] are showing that low prosthetic load materials, i.e., lightweight meshes with large pores, may be beneficial. Certain permanent synthetic meshes might be somewhat infection-resistant and therefore useful for permanent hernia repair in a contaminated environment. The presented results of this study seem to prove the advantages of macroporous mesh when used in clean-contaminated or contaminated field.

The authors speculate that the structure of the mesh allows for healing of the infection in the present experimental model. There was no persistent inflammation in the contaminated field when the mesh was properly implanted, i.e., spread flat to create a more favourable condition for the penetration of the immune system. Bending or folding the mesh resulted in the creation of dense texture “dead spaces”, making it difficult for immune cells to penetrate and leading to the persistence of the inflammatory process. This observation confirms the clinical findings of persistent abscesses and fluid reservoirs on explanted prostheses [36] and leads to the conclusion that many infections that are related to synthetic materials may be the result of a poor surgical technique.

The above study results, in the opinion of the authors, reinforce the view of the need for further clinical trials over a longer period of observation using the macropore monofilament meshes in a contaminated operative field as a valuable alternative to biological implants of unconfirmed value. It is also important to pay attention to the surgical technique, which can cause long-term infectious complications and even result in the need to remove the implants.

## Conclusions

A synthetic implant (monofilament macroporous) correctly placed in the operative field does not affect the normal healing process and the persistence of bacterial peritonitis in an experimental model. Inadequate surgical technique, poor positioning of the mesh resulting in rolling or corrugations, may favour the persistence of infection and sometimes lead to the need to explant the grid; thus, this potentially important factor requires attention to avoid a poor outcome.
